# Adult Nesidioblastosis in Chronic Kidney Disease

**DOI:** 10.1155/2019/7640384

**Published:** 2019-02-14

**Authors:** Eduardo Lozano-Melendez, Mercedes Aguilar-Soto, Luis Eugenio Graniel-Palafox, Laura Elena Ceceña-Martínez, Rafael Valdez-Ortiz, Fabio Solis-Jimenez

**Affiliations:** ^1^Faculty of Medicine and Nutrition of the Juarez University of the State of Durango, Durango, Durango, Mexico; ^2^Department of Internal Medicine, ABC Medical Center, Mexico City, Mexico; ^3^Department of Vascular and Interventional Radiology, General Hospital of Mexico ‘Dr. Eduardo Liceaga', Mexico City, Mexico; ^4^Department of Internal, General Hospital of Mexico ‘Dr. Eduardo Liceaga', Mexico City, Mexico; ^5^Department of Nephrology, General Hospital of Mexico ‘Dr. Eduardo Liceaga', Mexico City, Mexico

## Abstract

**Context:**

Nesidioblastosis is a rare cause of hyperinsulinemic hypoglycemia in adults. The diagnosis is further complicated in patients with kidney failure, since impaired renal function can cause hypoglycemia by itself and diagnostic criteria for this clinical scenario have not been developed yet.

**Case Description:**

We present the case report of a 36-year-old patient with end stage chronic kidney disease who presented to the emergency department because of hypoglycemia. However, the patient's hypoglycemia did not respond well to medical treatment; the diagnosis of hyperinsulinemic hypoglycemia was made due to the presence of inappropriately high levels of insulin, proinsulin, and C-peptide during an episode of hypoglycemia. Imaging studies were performed without any conclusive findings; so selective intra-arterial pancreatic stimulation with hepatic venous sampling (SACTS) was done. Based on the results of this study the patient was referred for subtotal pancreatectomy. Classic criteria for the diagnosis of insulinoma with SACTS required a 2-fold increase in insulin levels but newer criteria suggest thresholds that are useful in the differential diagnosis of insulinoma and nesidioblastosis. In our patient, the former criteria were positive; however, the new criteria were not compatible with insulinoma but with nesidioblastosis, which was the final histopathological diagnosis.

**Conclusion:**

This seems to be the first case report of a patient with end stage chronic kidney disease and nesidioblastosis, as well as the first case of hyperinsulinemic hypoglycemia in the context of kidney failure diagnosed by SACTS. We consider this method to be very useful in patients with renal impairment because peripancreatic insulin levels do not depend on the renal function.

## 1. Introduction

Laidlaw first used the term nesidioblastosis in 1938, to describe the formation of new islets of Langerhans from the ductal epithelium [1]. Nesidioblastosis is a frequent cause of hyperinsulinemic hypoglycemia in newborns, caused by specific mutations that promote a constant and unregulated secretion of insulin [2]. In adults, hyperinsulinemic hypoglycemia is mostly caused by insulinomas, while nesidioblastosis is much more infrequent. The first description of a nesidioblastosis case in adults was done in 1975 and since then only about 100 cases have been reported [3, 4]. Nesidioblastosis corresponds only to 4% of hyperinsulinemic hypoglycemia cases in adults because it is scarcely reported [5]. In addition, there are some conditions that complicate the approach of a patient with hyperinsulinemic hypoglycemia. One example is renal failure, which modifies the half-life of biomarkers, complicating its interpretation. This is further exemplified by the fact that only 4 cases of insulinoma have been reported in patients with renal failure [6]. We present the first case report of a patient with end stage chronic kidney disease, diagnosed with nesidioblastosis.

## 2. Case Report

A 36-year-old male patient with a 6-year history of chronic kidney disease of unknown etiology was brought to the emergency department due to neurological impairment that started during the previous hour characterized by altered mental status while he was walking down the street. On examination he was stuporous, with poor response to external stimuli. The patient was admitted to the hospital and vital signs and capillary glucose were determined. Glucose levels were 20 mg/dl and increased to 42 mg/dl after a 50 ml infusion of 50% dextrose. During his stay his mental status recovered after glucose levels were returned to normal parameters, requiring high doses of intravenous glucose. After his stabilization he was transferred to the internal medicine department. We confirmed that the patient was not taking medications that would cause hypoglycemia. On physical examination he was somnolent and pale, with slight oedema in both legs. A new episode of symptomatic hypoglycemia was observed while he was receiving an infusion of 20% dextrose. The infusion rate at that moment was 10.416 ml/hr. The infusion was not being weaned off or was an acute disruption. It caught our attention that although the patient was on a 20% dextrose infusion, he continued with hypoglycemia. Blood samples were taken and the results were abnormal (Table 1).

**Figure 1:**
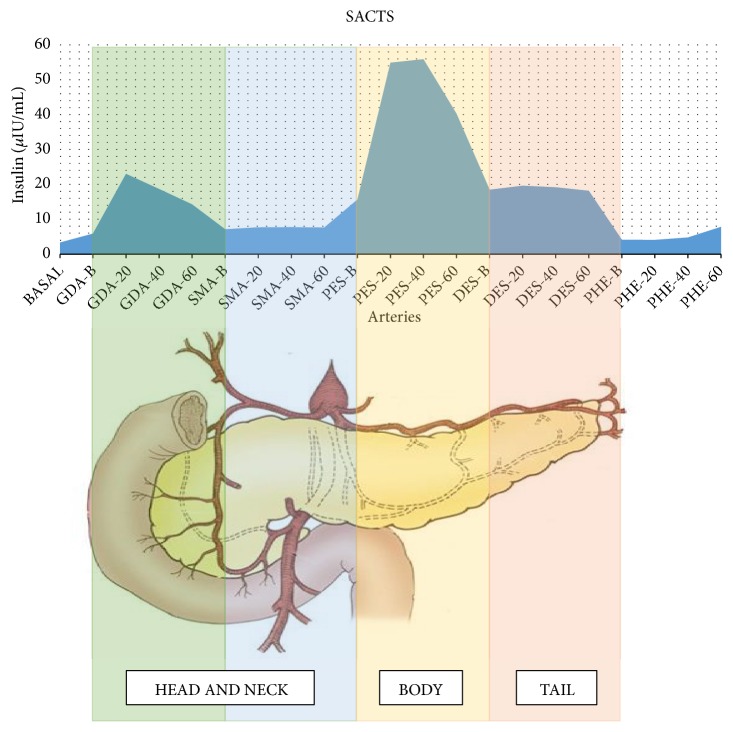
*Anatomical Relationship Between Areas and Arteries of the Pancreas Stimulated With Calcium.* Relationship of the main arteries of the pancreas and the areas that irrigate: gastroduodenal (GDA), superior mesenteric (SMA), and splenic artery. The splenic artery was divided into proximal (PES) and distal (DES) to differentiate the tail of the body. The proper hepatic artery was included to rule out tumor activity in the liver (PHE). The numbers indicate the seconds at which the sample was taken after the calcium injection. This suggests the most likely localization of the lesions.

**Figure 2:**
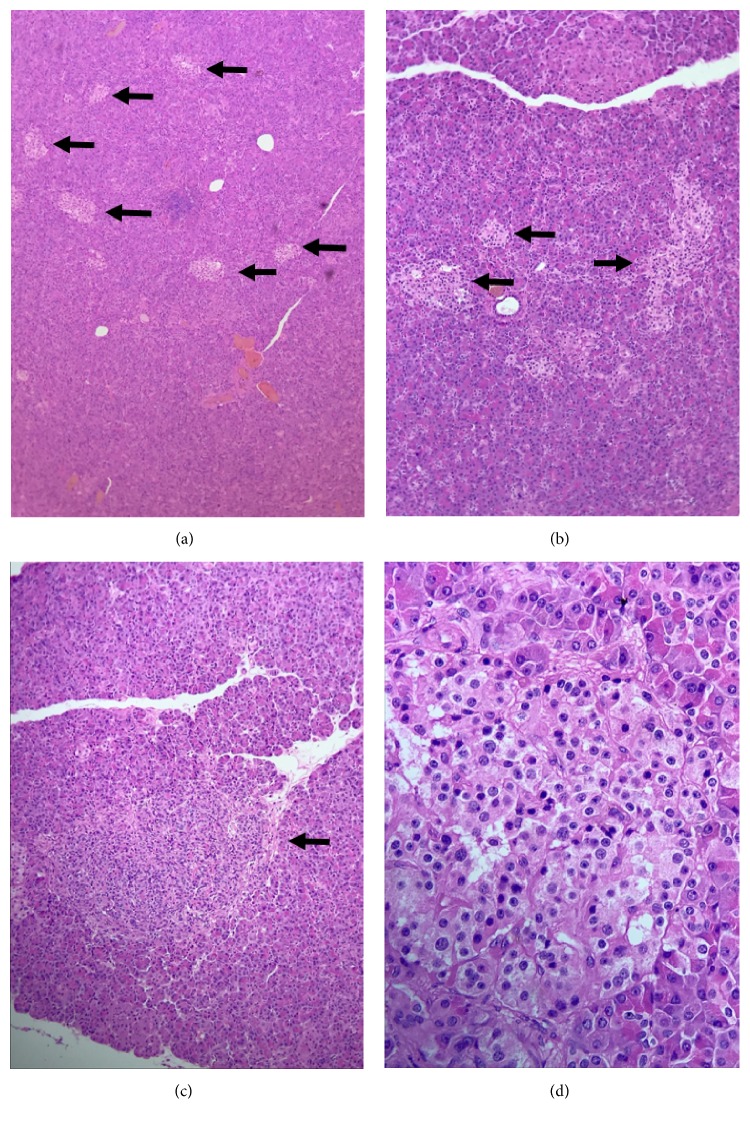
*Hematoxylin and eosin stain of cell-block preparations from biopsy specimen.* (a) Several clusters of *β* pancreatic cells are shown (arrows) (10x). (b) The typical cluster distribution near blood vessels is depicted (10x). (c) Pancreatic islets vary in size; very large islets are shown (arrow) (10x). (d) Hematoxylin and eosin stain shows cells with atypical morphology characterized by prominent nuclei and abundant granulated eosinophilic cytoplasm (40x).

Although hypoglycemia could be explained by chronic kidney disease, the diagnosis of insulinoma was considered, so a computed axial tomography with double contrast was taken but unfortunately there were no abnormal findings. During the patient's hospital stay he received several treatments that failed to achieve proper glucose control. We used ascending doses of diazoxide up to 600 mg/day with a poor response. In order to reduce episodes of hypoglycemia, we started with low doses of octreotide and found a good response that created tolerance quickly, so we decided to increase the dose by 0.1 mcg/kg/h always observing the same phenomenon. We decided to suspend this treatment when we reached 0.4. mcg/kg/h. Finally, we decided to maintain a continuous infusion of 50% dextrose with which we achieved serum glucose levels between 120 and 160 mg/dl. Magnetic resonance imaging and an endoscopic ultrasonography were performed but no conclusive data on any structural pancreatic disorder were obtained. In order to locate the tumor, we performed selective intra-arterial pancreatic stimulation with hepatic venous sampling at 0, 20, 40, and 60 seconds. High insulin levels were obtained after a selective injection of 0.025mEq/Kg calcium gluconate in the proximal splenic and gastroduodenal arteries (Table 2) (Figure 1). With these results, the patient was scheduled for surgery. During the procedure, bimanual palpation of the pancreas was performed, as well as a pancreatic ultrasound in which no tumor could be identified. The body and tail of the pancreas were resected. For two days the patient had an adequate glycemic control but after a couple of days, he presented with hypoglycemia again. The macroscopic pathology report did not show any tumor compatible with insulinoma; however, on microscopic examination pancreatic islets with elongated cells and clear cytoplasm compatible with nesidioblastosis were seen (Figure 2). Unfortunately, during his stay at the intensive care unit, the patient developed late-onset hospital-acquired pneumonia and, in spite of treatment, he developed sepsis followed by septic shock which ultimately caused his death.

## 3. Discussion

Current guidelines on the approach to the patient with hypoglycemia suggest that diagnostic studies should be done only in patients that present Whipple's triad which is characterized by hypoglycemia, symptoms of neuroglycopenia, and improvement of these symptoms when glucose is administered [7]. Nevertheless, the study of the patient with hypoglycemia should always be individualized in order to achieve a certain diagnosis [8]. Certain conditions, like kidney failure, are associated with an increased risk of hypoglycemia. Patients with impaired renal function have an increased incidence of both hypoglycemia and mortality from hypoglycemia [9]. The biological rationale for this is that the kidneys play an important role in the elimination of insulin from the systemic circulation. Because of the low molecular weight of insulin, it freely filters into the glomerulus and is then reabsorbed widely by the proximal tubule. Of the total renal clearance of insulin, approximately 60% is produced by glomerular filtration and 40% by extraction of peritubular vessels. In patients with advanced renal failure, baseline plasma levels of insulin, proinsulin, and C-peptide are elevated. As renal failure progresses, peritubular insulin uptake increases. This compensates for the decrease in degradation of filtered insulin as the glomerular filtration rate (GFR) decreases to less than approximately 20 ml / min, after which the clearance of insulin decreases, and its half-life increases. There is evidence from studies in animals that renal failure suppresses insulin metabolism in extrarenal sites such as skeletal muscles and liver. Some patients with end-stage renal disease have hypoglycemia due to prolonged persistence of circulating insulin, diet alterations, and exercise patterns [10]. A reduction in renal volume has also been associated with a decreased counterregulatory hormonal response and impaired renal gluconeogenesis [11]. We discarded this possibility, as there are large series of patients, which show that the vast majority of these cases are related to additional causes such as medication intake, sepsis, or severe malnutrition and not only to the CKD by itself [12]. On the other hand, there is also the possibility that some of the pathophysiological mechanisms by which hypoglycemia occurs improve considerably once renal replacement therapy is established, as in the case of our patient [13].

Our main suspicion was endogenous hyperinsulinism, so in order to confirm the diagnosis insulin, C- peptide, proinsulin, and *β*-hydroxybutyrate were measured. The interpretation of these tests in patients with renal impairment has not been defined and pancreatic polypeptides might be increased because of the renal disease itself and not because of endogenous hyperinsulinism [14].

Guidelines suggest the following criteria for the diagnosis of hyperinsulinemic hypoglycemia: serum glucose lower than 55 mg/dl (3.0 mmol/l), insulin of at least 3 *μ*IU/ml (18 pmol/l), C-peptide equal to or greater than 0.6 ng/ml (0.2 nmol/l), and proinsulin of at least 5.0 pmol/l with *β*-hydroxybutyrate of 2.7 mmol/L or less. Our patient fulfilled the five criteria [8]. No prolonged fasting was needed in this patient since he persisted with symptomatic hypoglycemia. The only step missing in our approach, according to the hyperinsulinemic hypoglycemia guidelines proposed by the endocrinology society was measurement of anti-insulin antibodies. Anti-insulin antibodies were not requested due to the strong ethnic component reported in the literature. These antibodies typically develop in Japanese and Korean patients and with less frequency in Caucasians. Besides, persons with this disorder often have a history of autoimmune disease or exposure to sulfhydryl-containing drugs [8]. Both situations were absent in the patient's medical history. It is also important to note that the patient has no history of diabetes; this is important because there are some studies that show that up to 20% of patients diagnosed with insulinoma have a history of diabetes [15]. On the other hand, it is less frequent to find patients with diabetes who later develop nesidioblastosis. Only few case reports give proof of this [16].

To our knowledge, there are no established criteria for the diagnosis of hyperinsulinemia in the context of chronic kidney disease. There exist only four cases reported of patients with hyperinsulinemic hypoglycemia and chronic kidney disease that we are aware of. After diagnostic evaluation, the four of them were found to be insulinomas [6, 14, 17, 18].

Based on what had been done with previous cases of insulinoma and chronic kidney disease, we started the protocol for tumor localization with imaging studies. However, there were no conclusive findings, so selective arterial calcium stimulation with hepatic sampling (SACTS) with radiological guidance was performed.

The original criteria for SACTS stated that a twofold increase at any sampling time or more on the basal insulin level should be considered as a positive response [19]. Thompson et al. suggested more stringent criteria for SACTS in order to distinguish insulinoma from nesidioblastosis. Considering that insulinomas have a higher response to calcium stimulation, they demonstrated that insulin values higher than >91.5 and >263.5 *μ*IU/mL had a specificity of 95% and 100%, respectively. They also demonstrated that a 19-fold increase in the basal value of insulin was 99% specific for the diagnosis of insulinoma [20].

Since our patient had a positive SACTS but did not fulfill the criteria suggested by Thompson et al., we considered nesidioblastosis as a diagnosis. Considering the low incidence of this condition, no large clinical trials for treatment evaluation have been conducted; thus surgical treatment remains controversial. Some authors suggest that an initial conservative surgery should be performed and subsequent surgeries are considered if symptoms persist [21].

One of the most difficult steps in this case was to decide whether partial or total pancreatectomy would be performed. After a multidisciplinary session, it was concluded that, given the undesired side effects of total pancreatectomy and the impact on the patient's life quality, it would be worth evaluating the functional status of the patient after performing a partial pancreatectomy. This decision was made based on the few data that exist about surgical treatment of this disease, which state that resection of 60-89% of pancreas (i.e., distal or subtotal pancreatectomy) is possibly the most appropriate surgery for nesidioblastosis because the risk of diabetes mellitus is below 10% with a 70% success rate in achieving normoglycemia [22]. Only if the patient persisted with hypoglycemia after the surgery, would a second intervention be planned in order to remove the rest of the pancreas.

The patient was referred for a subtotal pancreatectomy. Diagnosis of nesidioblastosis was confirmed by histopathology.

There will always be the possibility that pathological findings are not associated with hyperinsulinism. However, our strongest argument to demonstrate that our patient's hypoglycemia was due to a state of insulin hypersecretion is the calcium stimulation test. In it we found that the increased levels of insulin after administration of calcium are located in specific parts of the pancreas. Same areas show normal insulin levels before stimulation, and a few continue to do so even after stimulation. This indicates that although peripheral levels of insulin may be elevated as part of the decrease in their clearance, as a consequence of chronic kidney disease, the previously described findings suggest that the pancreas does indeed have an alteration that conditions insulin hypersecretion, since a normal pancreas does not secrete insulin when stimulated with calcium.

Exploration of the postmortem pancreas in order to rule out the diagnosis of insulinoma was not performed because the patient's family denied the procedure; however, the probability that the insulinoma was present was extremely low since during and before performing the pancreatectomy, bimanual palpation of the pancreas and pancreatic ultrasonography were performed, which report a sensitivity of up to 94.7% and 97.6%, respectively, in localizing an insulinoma.

Both findings for insulinoma were negative [23].

To the extent of our knowledge, this is the first case of nesidioblastosis reported in a patient with chronic kidney disease, as well as the first case of hyperinsulinemic hypoglycemia in the context of kidney failure diagnosed by SACTS. We consider this method to be very useful in patients with renal impairment because insulin levels are not modified by the factors mentioned earlier.

## Figures and Tables

**Table 1:** * Patient's β pancreatic polypeptides and reference values.* Reference values from the Endocrine Society Clinical Practice Guidelines for the Evaluation and Management of Adult Hypoglycemic Disorders.

Variable	Patient Values	Reference Values
Glucose	43 mg/dL	< 55 mg/dL
Insulin	25 *μ*IU/ml	> 3 *μ*IU/ml
C-Peptide	10.7 ng/dL	> 0.6 ng/dL
Proinsulin	50.7 pmol/L	> 5 pmol/L
*β*-hydroxybutyrate	0.1 mmol/L	< 2.7 nmol/L

**Table 2:** * Selective intra-arterial pancreatic stimulation with hepatic venous sampling (SACTS).* In the first column we find each artery from which a sample was collected. In each artery, before injecting calcium gluconate, a basal sample of insulin and glucose was taken. We can observe how during the study, glucose levels remained stable. Gastroduodenal (GDA), upper mesenteric (SMA), proximal splenic (PES), distal splenic (DES), and appropriate liver (PHE). Units of measurement: glucose mg/dL - Insulin *μ*IU/ml.

Artery	Basal Glucose	Basal insulin	T 20 Insulin	T 40 Insulin	T 60 Insulin
GDA	146	5.86	23.03	18.64	14.26
SMA	142	7.12	7.65	7.74	7.63
PES	144	15.64	54.9	55.9	40.27
DES	138	18.46	19.61	19.15	18.15
PHE	144	4.12	4.09	4.79	7.84
